# Distal thoracic oesophageal perforation secondary to blunt trauma: Case report

**DOI:** 10.1186/1749-7922-2-8

**Published:** 2007-03-21

**Authors:** Dirk C Strauss, Ruchi Tandon, Robert C Mason

**Affiliations:** 1 Department of General Surgery, St. Thomas' Hospital, Lambeth Palace Road, London SE1 7EH, UK

## Abstract

**Background:**

Traumatic perforation of the distal oesophagus due to blunt trauma is a very rare condition and is still associated with a significant morbidity and mortality. This is further exacerbated by delayed diagnosis and management as symptoms and signs are often masked by or ascribed to more common blunt thoracic injuries.

**Case report:**

We present a case of a distal oesophageal perforation, secondary to a fall from a third storey window, which was masked by concomitant thoracic injuries and missed on both computed tomography imaging and laparotomy. The delay in his diagnosis significantly worsened the patient's recovery by allowing the development of an overwhelming chest sepsis that contributed to his death.

**Conclusion:**

Early identification of an intrathoracic oesophageal perforation requires deliberate consideration and is essential to ensure a favorable outcome. Treatment should be individualised taking into account the nature of the oesophageal defect, time elapsed from injury and the patient's general condition.

## Background

Oesophageal perforation may result from iatrogenic, penetrating or blunt trauma. Most are secondary to oesophageal instrumentation and occur predominantly in the cervical oesophagus. Penetrating trauma accounts for 20–25%. Blunt trauma accounts for less than 10% of all oesophageal injuries. Perforation of the intrathoracic oesophagus is an extremely rare event [1,2], with an incidence at less than 0.2% [2-4]. The most recent review by Monzon et al found only sixteen cases in the world literature [5].

Oesophageal perforation is a serious injury to the gastrointestinal tract. Mortality rates of between 5–30% [6-8] are worsened by a delay in diagnosis: treatment after 24 hours can increase mortality to up to 50% [9,10].

The mechanism of thoracic oesophageal perforation in blunt trauma is unclear. The most commonly accepted theory, similar to the mechanism in Boerhaave syndrome, is an increase in intraluminal pressure against a closed glottis. This results in a tear at the weakest point of the oesophageal wall; usually the distal third of the oesophagus on the left. Other theories include; disruption of the oesophageal blood supply resulting in ischemia and late perforation, or a blast effect caused by a concomitant tracheal injury. Direct injury may also result from thoracic spine fractures or compression between the sternum and thoracic spine, as observed in high-speed road traffic accidents [5,11-14].

We outline the case of a patient who suffered a distal oesophageal rupture and discuss the pitfalls of management.

## Case Report

**Figure 1:**
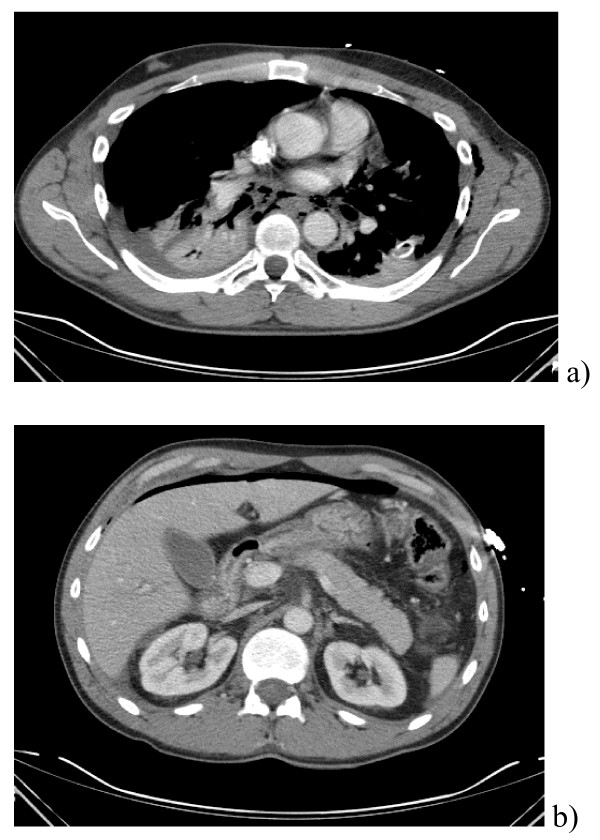
A) CT scan demonstrating pneumomediastinum. B) CT scan of abdomen demonstrating free intra-peritoneal air.

A 28 yr old male jumped from his burning apartment on the third floor. On admission he was resuscitated according to ATLS. On examination he was conscious with chest compression tenderness, a diffusely tender abdomen and bilateral lower limb fractures. Cervical spine, lumbar spine, pelvis and calcaneal films were all normal. A computed tomography (CT) chest and abdomen revealed bilateral pneumothoraces, air and haematoma within the mediastinum but no signs of a thoracic aorta rupture (Figure 1A and 1B). A significant amount of intraperitoneal air was noted. Following resuscitation and bilateral thoracotomy tubes he was taken to theatre for a urgent laparotomy. Despite a thorough search by an experienced surgeon no hollow viscus injury was found. Post operatively in ITU the patients condition continued to deteriorate. A repeat CT scan showed progressive extensive mediastinitis. An upper gastrointestinal endoscopy was performed. This revealed bilateral full thickness tears at the lower end of the oesophagus. The patient was then taken to theatre for thoracotomy and re-laparotomy Extensive lacerations to the distal oesophagus were identified. The degree of mediastinitis and extent of oesophageal injury precluded primary repair. An oesophagectomy and cervical oesophagostomy was performed. A decompressive gastrostomy and feeding jejunostomy were fashioned. A third procedure to washout his mediastinum and re-site chest drains before his condition stabilised. Five days later, he deteriorated suddenly; fresh bleeding was observed via his chest drains and despite an emergency thoracotomy the patient died. The autopsy showed a pericardiac tamponade.

## Discussion

Patients with oesophageal perforation may present with a constellation of symptoms including; dysphagia, odynophagia, chest pain, and dyspnoea. Tachycardia, pyrexia, worsening surgical emphysema and progressive sepsis may follow [11,12]. This case was unusual in that free intraperitonal air was identified in conjunction with a pneumomediastinum and bilateral pneumothoraces. The thoracic findings were attributed to his bilateral rib fractures and/or lung tauma. A laparotomy was performed to identify an intra-abdominal hollow viscus perforation. When no obvious perforation was identified at laparotomy the free intraperitoneal air was attributed to passage of air from the pleural space into the abdomen via anterior and posterior trans-diaphragmatic pathways [15,16].

For the diagnosis of oesophageal rupture the investigation of choice is a water-soluble contrast swallow. This requires a cooperative patient. Combative patients or those requiring intensive support and ventilation are ill-suited. Moreover under these conditions 10–40% of contrast swallows give a false-negative result [8,17,18]. Thoracic CT can identify the sequelae of oesophageal perforation, identified by an area of oesophageal wall thickening and or mediastinal air, but this is often obscured by oedema or haemorrhage [12] Mediastinal air is not uncommon in the polytrauma patient. CT is the most accurate method for illustrating para-oesophageal manifestations of rupture such as mediastinal collections, abscesses and effusions [17,19,20]. CT is a poor investigation to detect the presence or site of an oesophageal rupture. When a swallow is technically impossible or where a high index of suspicion remains despite a negative oesophagogram, flexible oesophagoscopy should be used. This is a widely available investigation allowing direct visualisation of the oesophageal mucosa. A sensitivity of 70–100% and a specificity of 96% [17] with a morbidity rate of only 0.2% [21,22]. It is thus a safe and effective method for both detection and exclusion of suspected oesophageal trauma.

Management options for oesophageal injuries are conservative or surgical and are summarised in Table 1.

**Table 1:** Management options for oesophageal perforation

**Clinical picture**	**Management**	**Reference**
Delayed presentation with minimal or no sepsis	Non-operative Monitor in high care facility Resuscitation I/V antibiotics Parenteral or jejunal nutrition	23
Small perforation	Tissue buttressing Mediastinal debridement I/V antibiotics Parenteral or jejunal nutrition	6,24,25,26,27
Large perforation	Oesophageal T tube Mediastinal debridement I/V antibiotics Parenteral or jejunal nutrition	24
Large perforation and extensive contamination	Cervical oesophagostomy Decompressive gastrostomy Gastro-oesophageal stapling Feeding jejunostomy I/V antibiotics	24,28
Perforation with pre-existing oesophageal disease or severely damaged oesophagus	Oesophagectomy	28

In this case the degree of mediastinal sepsis and extent of oesophageal injury precluded primary repair. Resection of the severely damaged oesophagus and cervical oesophagostomy was deemed the most appropriate treatment. However, the patient died from multi-organ failure secondary to overwhelming sepsis, of which a contributing factor must have been the delay in diagnosis of his oesophageal injury.

## Conclusion

Rupture of the oesophagus due to blunt trauma is rare. Intraperitoneal air following blunt trauma cannot be ignored. On table endoscopy at the first operation would have been diagnostic. It is vital to achieve an early diagnosis to limit thoracic contamination and improve survival.

## Competing interests

The author(s) declare that they have no competing interests.

## Authors' contributions

DCS Collected data, literature review and drafted the original manuscript

RT Collected data and drafted the original manuscript

RCM Conceived idea for manuscript and acts as guarantor of manuscript

All authors read and approved the final manuscript.
